# The Driving Mechanism of Phytoplankton Resource Utilization Efficiency Variation on the Occurrence Risk of Cyanobacterial Blooms

**DOI:** 10.3390/microorganisms12081685

**Published:** 2024-08-16

**Authors:** Yongxin Zhang, Yang Yu, Jiamin Liu, Yao Guo, Hongxian Yu, Manhong Liu

**Affiliations:** 1 College of Wildlife and Protected Area, Northeast Forestry University, Harbin 150040, China; yongxinzhang2023@163.com (Y.Z.);; 2 China Geological Survey Harbin Natural Resources Comprehensive Survey Center, Harbin 150081, China; 18182803312@163.com

**Keywords:** phytoplankton, Cyanobacteria, resource utilization efficiency, nutrients, generalized additive model

## Abstract

Algae are highly sensitive to environmental factors, especially nutrient fluctuations; excessive nutrients can lead to the proliferation of specific algae species, resulting in dominance. In this study, we aimed to reevaluate changes in algal dominance from the perspective of resource utilization efficiency (RUE). We established 80 monitoring sites across different water systems, collecting water and phytoplankton samples. Using canonical correspondence analysis (CCA) and a generalized additive model (GAM), we analyzed the correlation between phytoplankton RUE and nutrient concentrations, quantifying the corresponding relationship between algal dominance and RUE. Our results indicate a significant negative correlation between the RUE of total phosphorus (TP) and total nitrogen (TN) concentration, but a positive correlation with N:P. The RUE of TN was negatively correlated with TN concentration and N:P. We constructed GAMs with interaction terms and confirmed a nonlinear relationship between algal dominance and RUE. When the RUE of TN was low, a positive correlation was observed, while a negative correlation was observed otherwise. These findings reveal the ecological adaptability of algal communities and provide valuable insights for predicting the risk of algal bloom outbreaks.

## 1. Introduction

The frequent occurrence of cyanobacterial blooms has become a serious challenge for freshwater ecosystems worldwide [1,2], particularly in rivers of agricultural areas. Excessive nutrient inputs have led to increased dominance of cyanobacteria, posing a serious threat to water quality and ecological balance [3]. The mechanisms behind cyanobacterial formation and their impacts on ecosystem structure and function are complex and variable [4,5,6] and may exhibit seasonal variations. Resource utilization efficiency (RUE) is a new indicator that can interpret the extent of cyanobacterial blooms [7]. Research on the relationship between cyanobacterial dominance and phytoplankton RUE is increasingly significant, especially from the perspective of seasonal changes in ecosystems [8,9]. It has been demonstrated that there is a nonlinear relationship between phytoplankton RUE and algal community composition [10]. Our aim is to elucidate the nonlinear nature of this relationship, which supports early warning of harmful algae [11].

When cyanobacterial communities dominate, the structure and function of phytoplankton communities are severely disrupted [12]. The dominance of cyanobacteria is regulated by numerous factors and significantly correlates with nutrient concentrations, temperature, and N:P [13]. Cyanobacterial dominance is closely linked to their physiological mechanism [14]. Additionally, cyanobacteria can achieve higher dominance due to changes in phytoplankton RUE or environmental conditions [15], indicating a relationship between cyanobacterial dominance and phytoplankton RUE [16]. Both cyanobacterial dominance and RUE are influenced by nutrient concentrations [17]. With the progression of urbanization and excessive nutrient discharge, there is mounting evidence that the risk of cyanobacterial blooms is increasing, which significantly impacts phytoplankton community structure and resource utilization efficiency [18,19]. Human activities have greatly altered the relationship between phytoplankton diversity and ecosystem function [20]. The relationship between nutrient concentrations and phytoplankton RUE is intricate and can also be influenced by other environmental factors, particularly water temperature, hence our seasonal study [21]. Historical research indicates that the phytoplankton community structure is linked to RUE [22], which demonstrates a nonlinear relationship [23]. Therefore, we aim to reveal the risk of cyanobacterial blooms from the perspective of RUE.

For studying the mechanisms affecting algal blooms, remote sensing is currently a popular approach for macroscopic research [24,25,26]. However, it cannot analyze the occurrence mechanisms of algal blooms at a smaller scale. Regardless, molecular monitoring as a new form of early-warning method has opened up new perspectives for studying algal blooms [11]. More importantly, phytoplankton are sensitive to changes in environmental factors such as nutrients [27]. Revealing the coupling relationship between algal blooms and water environmental factors is extremely important, especially regarding nutrients in the water [28,29,30]. Therefore, adopting appropriate methods to study algal blooms and developing reasonable management strategies is an urgent global task [31]. Various research techniques have been employed to uncover the relationships between nutrients and phytoplankton in aquatic ecosystems. Canonical correspondence analysis (CCA) is a widely utilized and robust method for analyzing the correlations between environmental factors and biological communities [32]. Additionally, various techniques and models have been employed to study algal blooms, such as the partial least-squares structural equation model [33] and the Bayesian method [34,35]. Generalized additive models (GAMs) have demonstrated efficacy in elucidating the intricate interplay between species diversity and ecosystem functionalities [36,37]. By integrating river sample data with GAMs to analyze the relationship between cyanobacterial dominance and phytoplankton resource use efficiency, we can enhance our understanding of this issue [38]. Through comparative analysis of seasonal data, we aim to elucidate the underlying mechanisms driving cyanobacterial blooms and discern the seasonal dynamics influencing them. This approach will yield scientific insights and management strategies to mitigate the challenge posed by cyanobacterial blooms.

Through analysis of river samples, we hypothesize a complex and nonlinear relationship between cyanobacterial dominance and phytoplankton RUE. The mobility and seasonal variations of rivers may lead to continuous changes in environmental factors [39], thereby influencing the formation of cyanobacterial blooms and phytoplankton RUE. Our study was conducted across four distinct water systems characterized by diverse hydrological conditions: a typical urban river, a natural river within a nature reserve, a natural lake, and a wetland water system. Located in cold regions with prolonged winter ice cover, we conducted sampling during spring, summer, and autumn. The research encompassed four main phases: (1) establishment of monitoring stations: setting up monitoring stations to assess nutrient levels and phytoplankton communities, with subsequent analysis of their structure; (2) spatiotemporal analysis: investigating variations over space and time in nutrient concentrations and cyanobacterial dominance; (3) CCA analysis: utilizing CCA to explore relationships between phytoplankton RUE and nutrient levels; and (4) GAM construction: developing a GAM to examine the nonlinear dynamics between cyanobacterial dominance and RUE of nutrients, offering insights into mitigating the risk of cyanobacterial blooms.

## 2. Method

### 2.1. Study Locations

**Figure 1:**
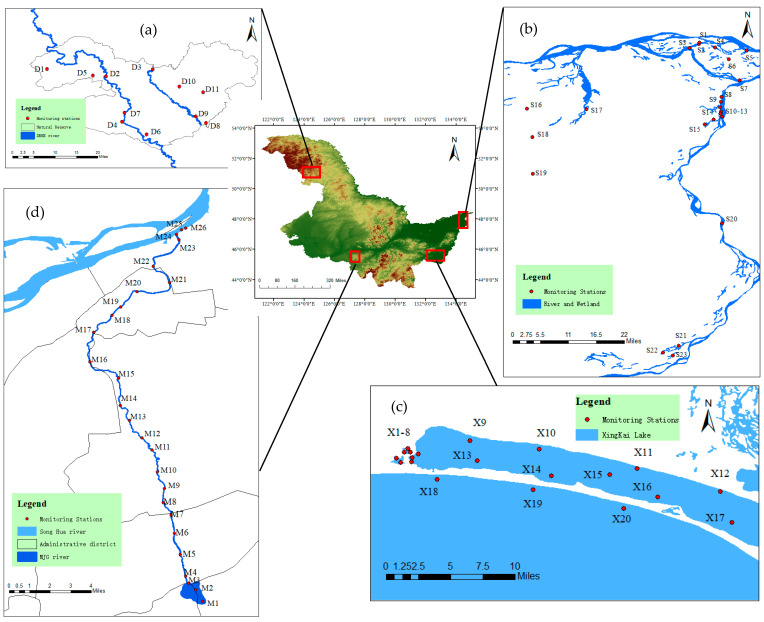
Spatial distribution of 80 phytoplankton and water monitoring stations. (**a**) D1–D11. (**b**) S1–S23. (**c**) X1–X20. (**d**) M1–M26.

This study selected four types of typical water bodies and established 80 monitoring stations based on the principle of uniform sampling. (1) The DBKE River, situated in the Duobu National Nature Reserve in China, encompasses a basin area of 5490 square kilometers. Spring and autumn are not distinct, with a short summer and large annual temperature variations, reaching up to 82.7 °C. The average yearly temperature ranges from −1.3 °C to 2 °C, with a minimum of −45.4 °C and a maximum of 37.3 °C. The annual average precipitation is 500 mm. The river flows through a water protection area primarily composed of forests, shrubs, and herbaceous wetlands, experiencing relatively low levels of human disturbance. Eleven monitoring stations were established, including stations on its tributaries (D1–D11, Figure 1a). (2) The Sanjiang National Nature Reserve and Heixiazi National Nature Reserve cover a combined area of 2105 square kilometers. Seasons are distinct, with a long freezing period and concentrated precipitation. The annual average temperature is 2.2 °C, and the yearly average precipitation is 603.8 mm. The region is primarily dominated by swamp wetlands, experiencing similarly low levels of human disturbance. Twenty-three wetland monitoring stations were established (S1–S23, Figure 1b). (3) The Xingkai Lake National Nature Reserve covers an area of 2246 square kilometers, featuring freshwater lakes and a small amount of swamp wetlands. The annual average temperature is 3.1 °C, with July being the warmest at 21.2 °C and January the coldest at −19.2 °C. The annual precipitation averages 750 mm, with about 70% falling in the summer. Twenty monitoring stations were established within the research area (X1–X20, Figure 1c). (4) The Majiagou River in Harbin City is a typical urban river, stretching 44.3 km. It flows through the main urban area of Harbin City and experiences relatively severe human disturbance. In the main urban area of Majiagou River, the annual average temperature is 3.5 °C, with January being the coldest at −19.4 °C and July the warmest at 22.8 °C. The annual precipitation averages 530 mm, mostly concentrated in July and August. Twenty-six monitoring stations were established (M1–M26, Figure 1d). All monitoring stations are located in northeast China, which is characterized by a cold temperate continental climate and long periods of ice cover in the rivers during winter.

### 2.2. Biological and Environmental Data Monitoring

This study was conducted during the spring, summer, and autumn of 2022–2023 to sample phytoplankton and water quality. To avoid the influence of light on phytoplankton sampling, all sampling was conducted during the daytime within the same time frame. Due to the numerous monitoring stations, sampling was divided across 2–3 days. On-site, a water quality analyzer (YSI 6600) was used to monitor water chlorophyll concentration. Water samples were collected from 0.5 m below the water surface using 1 L organic glass containers [40]. Additionally, 1.5% Lugol’s solution was added on-site to fix the phytoplankton [41]. After 48 h of settling in the laboratory, the supernatant was absorbed using a siphon method, and the sample volume was concentrated to 50 mL. Phytoplankton were identified and counted under a 10 × 40 optical microscope. Total nitrogen (TN) and total phosphorus (TP) concentrations were determined in the laboratory using alkaline potassium persulfate digestion-UV spectrophotometry and ammonium molybdate spectrophotometry, respectively [42].

### 2.3. Data Statistical Analysis and Modeling

#### 2.3.1. Phytoplankton RUE and Cyanobacterial Dominance

The resource use efficiency of N and P was calculated as the ratio of chlorophyll-a concentration or cyanobacteria density to the concentrations of TN and TP, respectively, using the following formula:

$$\mathrm{RUE}=\frac{\mathrm{Chl}-a(\mu g/L)}{\mathrm{TN}(\mathrm{mg}/L)\cdot \mathrm{or}\cdot \mathrm{TP}(\mathrm{mg}/L)}$$

Abundance is an effective indicator of the status of phytoplankton communities [43,44]. In this study, relative abundance was chosen to reflect the dominance level of cyanobacteria at each monitoring point.

#### 2.3.2. Data Analysis

To examine the variations in cyanobacterial dominance and nutrient resource use efficiency among the four types of water bodies, we conducted a one-way analysis of variance (ANOVA) followed by post hoc multiple comparisons using the LSD test. When the homogeneity of variances test resulted in a *p*-value less than 0.05, we employed the Kruskal–Wallis nonparametric test. Both the ANOVA and Kruskal–Wallis tests were performed using R.

To elucidate the variation in RUE along the gradient of nutrient concentration, redundancy analysis (RDA) was employed. The data were transformed using a log10(x + 1) transformation and subjected to detrended correspondence analysis. CANOCO 4.5 software was used for these analyses.

As neither the dominance of cyanobacteria nor their logarithms exhibited a normal distribution (*p* < 0.05), we opted for nonparametric analysis using GAMs. Minimal smoothness (k = 3) was employed to calculate the regressions of GAMs, with *p*-values (*p* < 0.05) reported from GAM regression summaries when the residuals demonstrated a normal distribution. GAMs were used to elucidate the relationship between the dominance of cyanobacteria and RUE. We employed the Akaike information criterion (AIC) to compare the goodness of fit of GAMs, considering multiple significant factors and interaction terms. The model with the best structure was identified, ensuring that residuals fitted or approximated a normal distribution. Finally, response curves for each smoothing term of the GAMs were fitted to ascertain the relationship between the dominance of cyanobacteria and RUE. The general structure of a GAM was as follows:

$$g(E(Y))=\beta_{0}+f_{1}{(x}_{1})+f_{2}{(x}_{2})+\cdots +f_{m}{(x}_{m})$$

Here, g(g(.)) is a link function, E(Y) is the expected value of the response variable, β_0_ is a constant, and f_i_(x_i_) is a smooth function. We used the “mgcv” package version 4.2.1of R Project to implement GAMs.

## 3. Result

### 3.1. Dominance of Cyanobacteria and RUE Analysis

**Figure 2:**
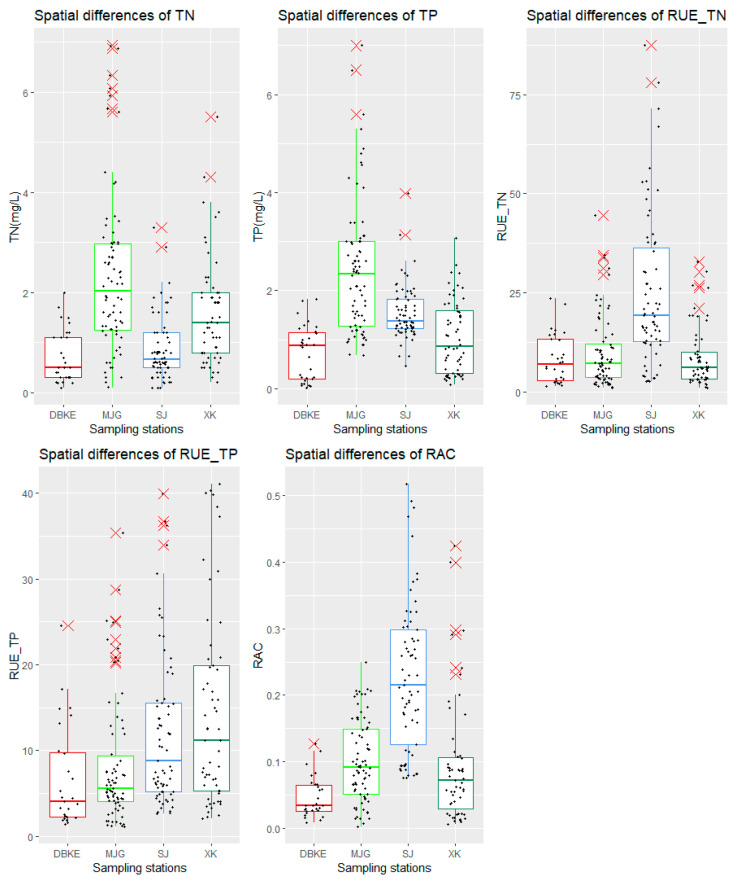
Spatial variations in nutrient concentrations, RUE, and cyanobacterial dominance (RAC). The upper edge of the box plot represents the upper quartile, the lower edge represents the lower quartile, and the middle line represents the mean; the dots represent the observed values.

The results indicated a close correlation between nutrient concentrations in water and the degree of human interference. As shown in Figure 2, the nutrient concentrations were highest in the typical urban river, Majiagou River, with average values of 2.32 mg/L for TN and 2.43 mg/L for TP. In the Sanjiang Wetland, water bodies exhibited higher TP concentrations compared with TN concentrations due to agricultural interference, with average values of 0.88 mg/L for TN and 1.54 mg/L for TP. The lowest nutrient concentrations were found in the DBKE River located in the northeastern forest area of China, with average values of 0.72 mg/L for TN and 0.73 mg/L for TP [45,46].

The average relative abundance of cyanobacteria in the Sanjiang Wetland water system was relatively high at 22.7%, posing a risk of algal blooms, mainly dominated by *Microcystis aeruginosa*, which was associated with agricultural development in the Sanjiang Plain. The average TN RUE was highest in the Sanjiang Wetland water system at 25.23, attributed to sufficient phosphorus resources leading to the higher RUE of nitrogen. The average value for other three types of water bodies was 9.35. The average RUE of TP was highest in Xingkai Lake at 14.62, while for the other three types of water bodies, it was 9.61.

### 3.2. The Spatiotemporal Variations in the Dominance of Cyanobacteria and RUE

First, variance homogeneity tests and Shapiro–Wilk tests were conducted to determine the appropriate method for one-way analysis of variance. The results indicated that the variance homogeneity tests for TN, TP, dominance of cyanobacteria, RUE-TN, and RUE-TP did not pass; thus, the more robust Welch’s ANOVA was employed. As shown in Table 1a, significant spatial differences were observed for each factor (*p* < 0.05). Post hoc multiple comparisons using the LSD method (Table 1b) revealed significant differences among seasons for TN, RUE-TN, and RUE-TP (*p* < 0.05). The TN concentration in spring was significantly higher than in other seasons, but the RUE values for TN and TP were significantly lower. No significant differences were observed among seasons for TP concentration and dominance of cyanobacteria.

### 3.3. RUE Changes along the Nutrient Concentration Gradient

**Figure 3:**
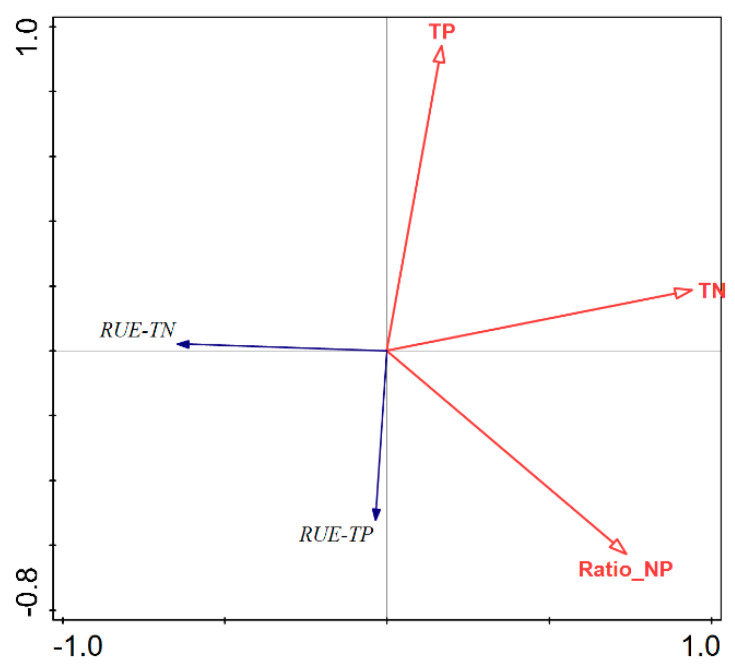
RDA results of RUE and environment factors.

After performing detrended correspondence analysis and assessing the gradient axis lengths, it became apparent that RDA outperforms CCA. Hence, we opted for RDA as the foundation of our study. As depicted in Figure 2, the length of the arrow signifies the correlation strength between environmental variables and phytoplankton, with a longer arrow denoting a stronger correlation. The vertical separation between various phytoplankton categories and the environmental variable axis indicates their correlation strength, with greater separation signifying a stronger correlation. According to the RDA analysis, TN showed the highest explanatory power for RUE, accounting for 21.6%, followed by the nitrogen-to-phosphorus ratio (Table 2). The RUE-TN and RUE-TP of phytoplankton were significantly negatively correlated with water nutrient concentrations, while RUE-TP showed a certain degree of positive correlation with the N:P. RUE-TN exhibited a negative correlation with the N:P (Figure 3).

### 3.4. Response of RUE to Cyanobacterial Dominance

In spring, summer, and autumn, we constructed GAMs (Model 1–3) to analyze the relationships between cyanobacterial dominance and RUE-TN, RUE-TP, and N:P, respectively. In spring, Models 1–3 explained 42.3%, 58.1%, and 70% of the deviance explained (DE), respectively. To improve DE, we introduced interaction terms (Models 4–5), resulting in a 31% and 31.3% increase in DE compared with Model 1. In summer, Models 1–3 explained 4.99%, 18.6%, and 17.9% of DE, respectively. By introducing interaction terms to enhance model variance explanation, Models 4–5 showed a DE increase of 13.31% and 15.91% over Model 1, respectively. In autumn, Models 1–3 explained 16.2%, 17.2%, and 16.2% of DE, respectively. After adding interaction terms, DE improved by 0.1% and 4.8%. The results indicated that adding interaction terms consistently increased DE to some extent (Table 3). We selected the models with the highest DE for further analysis of the relationships. The model’s robustness was verified through the histogram of the residuals. As shown in the Supplementary Material, the residuals essentially followed a normal distribution, indicating the model’s reliability.

**Figure 4:**
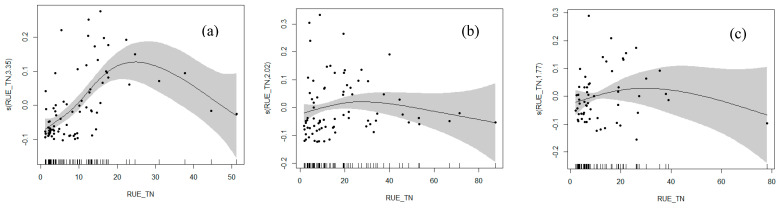
The response curves of cyanobacterial dominance and RUE: (**a**) Spring. (**b**) Summer. (**c**) Autumn.

The results from the GAM confirmed our hypothesis: nutrient utilization efficiency significantly impacts cyanobacterial dominance and exhibits a notable nonlinear relationship (Figure 4). The response of cyanobacterial dominance to RUE-TN across the three seasons showed both similarities and distinct differences. In spring, cyanobacterial dominance peaked when RUE-TN was around 24. Below this value was a clear positive correlation, while above it was a clear negative correlation. Similarly, in summer and autumn, cyanobacterial dominance reached its highest levels when RUE-TN was between 22 and 26. However, the correlation between cyanobacterial dominance and RUE-TN was weaker compared with spring. In both seasons, there was a weak positive correlation when RUE-TN was below the threshold and a weak negative correlation when it was above the threshold. Due to the poor accuracy and high error values in the GAM that was constructed for cyanobacterial dominance and RUE-TP, we decided not to proceed with further analysis.

## 4. Discussion

In our study, we strategically established monitoring stations across four distinct water body types to assess water quality and phytoplankton dynamics. We collected comprehensive samples and employed advanced analytical techniques, including CCA and GAMs, to reevaluate the risk of cyanobacterial blooms through the lens of RUE. This approach has yielded critical insights, offering a refined understanding and improved strategies for managing the risk of cyanobacterial blooms.

### 4.1. The Correlation between RUE and Nutrient Concentration

The study results indicated that the RUE of TP was significantly negatively correlated with TP concentration. Drawing from historical research conclusions, we postulate that elevated TP concentrations might induce shifts in the phytoplankton community structure, especially by fostering the proliferation of dominant species [47]. However, elevated RUE was linked to higher diversity levels [48]. Dominance by a single species often results in decreased diversity, and the dominant species may not be the most efficient at utilizing TP. Additionally, high TP concentrations can induce explosive growth of dominant species like cyanobacteria, exacerbating ecosystem instability [49]. This instability might reduce RUE, as the excessive growth of dominant species can impede the growth and resource utilization of other species. This phenomenon has been well documented in previous research [50]. In environments with high TP concentrations, phytoplankton might be in a state of overnutrition. We hypothesized that high TP concentrations can lead to excessive phytoplankton growth, and this excess biomass might not be efficiently utilized, resulting in decreased RUE. We observed a positive correlation between the RUE of TP and N:P, leading us to hypothesize that variations in the N:P significantly impact cyanobacterial dominance. When the N:P was low, indicating nitrogen limitation, cyanobacteria can often exploit TP more effectively, thereby increasing the RUE of TP. This efficiency arises from cyanobacteria’s superior ability to utilize TP resources under conditions of low N:P [51], leading to a positive correlation. Additionally, an appropriate N:P helped maintain nutrient balance in the ecosystem [52]. When the N:P is favorable, cyanobacteria and other phytoplankton can more efficiently utilize TP, thereby enhancing the RUE of TP [53]. Consequently, we hypothesize that the positive correlation between the N:P and the RUE of TP might indicate intricate interactions between nutrient elements within the ecosystem. Our research substantiated the historical conclusion that the N:P is a crucial determinant of cyanobacterial dominance [54].

The RUE of TN displayed a distinct negative correlation with TN concentration. Analogous to the mechanisms observed for TP, elevated TN levels often signal nutrient excess in the water, which can precipitate the unchecked proliferation of phytoplankton [29]. High TN concentrations led to the overutilization of nitrogen by phytoplankton, reducing the RUE of TN. However, when other nutrients, such as TP, were limiting, phytoplankton did not fully exploit nitrogen sources even with high TN levels. Our results showed an inverse relationship between the RUE of TN and the N:P. We hypothesized that this resulted from nutrient limitation; a low N:P indicated excessive nitrogen relative to phosphorus, making phosphorus the limiting factor; this aligned with previous research [55]. Additionally, the ecological niche adaptations of phytoplankton were influential. For instance, some species, including cyanobacteria, were well adapted to environments with a low N:P [56,57]. These species were more efficient at utilizing nitrogen in low N:P conditions, where phosphorus was less limiting. Consequently, in such environments, cyanobacteria can outcompete other species, reducing the RUE of TN.

### 4.2. The Coupling Relationship between RUE and Cyanobacterial Dominance

Across all three seasons, the relationship between cyanobacterial dominance and the RUE of TN exhibited a nonlinear pattern characterized by a peak. Below this peak, there was a positive correlation, while above it, the correlation turned negative. This nonlinear relationship was likely influenced by a combination of factors, including seasonal variations, aquatic environmental conditions, and the ecological and physiological traits of cyanobacteria [58]. Additionally, aquatic environmental conditions, which fluctuated with the seasons, likely influenced cyanobacteria’s ability to utilize nitrogen and their overall growth and reproduction capacities. For example, in spring and summer, the elevated water temperatures and intensified light conditions facilitated cyanobacterial growth, subsequently raising the peak of RUE. More importantly, the competitive dynamics among phytoplankton played a pivotal role. We hypothesized that when the RUE of TN was low, cyanobacteria might have had a competitive advantage over other phytoplankton, while high RUE could have favored the growth and reproduction of other phytoplankton species. However, this hypothesis awaited further experimental validation. Essentially, this relationship reflected the ecological adaptability and competitive capacity of cyanobacteria under varying RUE conditions.

Furthermore, we observed that the response relationship exhibited greater fluctuations in spring. This was likely due to variations in biological adaptability, leading to biomass peaks for certain phytoplankton species during this season [59]. Spring, following winter freeze-up, was a period of dramatic changes in aquatic environmental conditions, including water temperature, light intensity, and nutrient status. Furthermore, spring was a critical reproductive and growth period for many aquatic organisms, heightening competition among phytoplankton. This competition may have caused greater fluctuations in the response of cyanobacterial dominance to the RUE of TN. Additionally, the transitional nature of aquatic ecosystems in cold regions during spring led to significant changes in the structure and function of biological communities. This seasonal ecological dynamism likely contributed to the observed variability in cyanobacterial responses to the RUE of TN. Additionally, climate change, grazing pressure, and human disturbances were also potential factors influencing algal community dynamics. Climatic changes, such as temperature fluctuations, floods, and rainfall, along with grazing by herbivores like zooplankton, and human activities like agricultural runoff and pollution, all significantly impact phytoplankton dynamics. These factors can alter nutrient inputs, influence community structure, and drive changes in the RUE in freshwater ecosystems.

While our findings highlighted the influence of seasonal changes on cyanobacterial dominance, we acknowledged this study’s limitations. The variability in environmental conditions and competition dynamics might have introduced complexities not fully captured by our models. Future research should incorporate a broader range of environmental factors and longer temporal scales to better understand the intricate mechanisms driving cyanobacterial blooms. More urgently, we need to discuss the food web within the ecosystem. Variations in phytoplankton can impact zooplankton and, subsequently, higher trophic levels like fish, leading to cascading effects throughout the ecosystem. Understanding these interactions helps us grasp how shifts in phytoplankton influence overall ecosystem health and stability, guiding more effective management strategies.

### 4.3. Risk of Cyanobacterial Bloom Outbreaks

Our research findings substantiated the hypothesis that nutrient RUE is correlated with nutrient concentration. Additionally, cyanobacterial dominance demonstrated a nonlinear relationship with the RUE of TN. In studies of algal blooms in temperate and tropical regions, summer is typically considered the high-risk season for bloom outbreaks [60]. However, our study area is located in the cold regions of northeastern China, where rivers experience long periods of freeze-up during winter, resulting in unique hydrological conditions [61]. The response relationship in spring exhibited more significant fluctuations, and the peak dominance of cyanobacteria was higher. This suggests that the pronounced nutrient and environmental changes in spring significantly influenced the growth and reproduction of cyanobacteria, increasing the risk of bloom occurrence. Additionally, the melting of ice in spring introduced substantial amounts of nutrients, such as nitrogen and phosphorus, stored over the winter, stimulating the proliferation of cyanobacteria. Consequently, the risk of cyanobacterial blooms exhibited considerable variability, manifesting as rapid increases or decreases in cyanobacterial abundance. This conclusion requires further comprehensive validation in future studies. Although our study indicated a lower risk in summer compared with spring, it remained a peak period for bloom occurrences. Effective management strategies included rigorous inspections to detect early signs of blooms and the application of physical or chemical controls, such as aeration, ultrasound, or chemical treatments, to curb bloom growth [62,63].

### 4.4. Outlook for the Future

Due to methodological limitations, the model’s explanatory power was insufficient, preventing us from analyzing the coupling relationship between TP resource utilization and cyanobacterial dominance. Moreover, at a broader scale, climate is a significant factor influencing cyanobacterial bloom outbreaks [64,65]. In the future, we aim to establish a long-term phytoplankton monitoring database based on this study. This will enable us to analyze the correlation between climate conditions and cyanobacterial bloom risk, and investigate the mechanisms driving changes in bloom occurrence risk under current climate change scenarios.

## 5. Conclusions

This study concludes the following:

1. There is a correlation between the nutrient utilization efficiency and concentration of nutrients. The efficiency of TN utilization is negatively correlated with the concentration of TN and N:P. Similarly, the efficiency of TP utilization is negatively correlated with the concentration of TP but positively correlated with the N:P.
2. This study confirms the nonlinear relationship between the risk of cyanobacterial blooms and nutrient utilization efficiency, with similar patterns observed across the three seasons. The fluctuation in cyanobacterial dominance is most pronounced in spring.
3. When the efficiency of TN utilization is low, the risk of cyanobacterial blooms is positively correlated with the efficiency of TN utilization. Conversely, when TN utilization efficiency is high, the risk of cyanobacterial blooms is negatively correlated with TN utilization efficiency.

Our research provides insights into controlling the risk of cyanobacterial blooms, which can promote the ecological management of rivers.

## Figures and Tables

**Table 1:** (a) ANOVA Results: Spatial Variation Analysis. (b). ANOVA Results: Seasonal Variation Analysis.

**(a)**						
	**Sampling Region**				**Effects of Sampling Region**	
	**DBKE**	**MJG**	**XKH**	**SJ**	**Statistics**	***p*-Value**
TN	0.72 ± 0.52 ^c^	2.31 ± 1.6 ^a^	1.61 ± 1.06 ^b^	0.88 ± 0.65 ^c^	F = 24.587	<0.05
TP	0.73 ± 0.55 ^c^	2.43 ± 1.36 ^a^	1.06 ± 0.74 ^c^	1.54 ± 0.55 ^b^	F = 35.499	<0.05
RUE-TN	8.31 ± 6.2 ^b^	10.11 ± 9.02 ^b^	8.88 ± 7.56 ^b^	25.23 ± 19.28 ^a^	F = 26.113	<0.05
RUE-TP	6.72 ± 6.04 ^c^	8.24 ± 7.25 ^bc^	14.64 ± 11.55 ^a^	12.20 ± 9.15 ^ab^	F = 7.918	<0.05
Dominance	0.05 ± 0.03 ^c^	0.11 ± 0.06 ^b^	0.095 ± 0.093 ^b^	0.23 ± 0.112 ^a^	F = 45.833	<0.05
**(b)**						
	**Season**				**Effects of Season**	
	**Spring**	**Summer**	**Autumn**		**Statistics**	***p*-Value **
TN	1.86 ± 1.66 ^a^	1.25 ± 0.99 ^b^	1.54 ± 1.09 ^ab^		F = 6.900	<0.05
TP	1.52 ± 0.78	1.52 ± 1.08	1.79 ± 1.51		F = 1.843	0.161
RUE-TN	9.85 ± 9.48 ^b^	12.44 ± 10.3 ^a^	12.51 ± 12.14 ^b^		F = 8.097	<0.05
RUE-TP	8.25 ± 6.88 ^b^	13.00 ± 11.72 ^a^	10.93 ± 8.49 ^ab^		F = 6.650	<0.05
Dominance	0.11 ± 0.09	0.10 ± 0.07	0.14 ± 0.09		F = 2.551	0.08

The significant post hoc results (*p* < 0.05) from LSD test are indicated by different lowercase letters.

**Table 2:** Summary of RDA between RUE and environmental variables.

Factors	Explains (%)	Contribution (%)	Pseudo-F	*p*-Value
TN	21.6	60.4	58.0	<0.05
Ratio_NP	10.4	29.2	32.2	<0.05
TP	3.7	10.3	12.0	<0.05

**Table 3:** GAM of RUE and optimal structures (RAC: Cyanobacterial Dominance).

	Spring		Summer		Autumn	
GAM	Deviance Explained (DE)	DE Variation with Model 1	Deviance Explained (DE)	DE Variation with Model 1	Deviance Explained (DE)	DE Variation with Model 1
Model 1: RAC~s (RUE-TN)	42.3%		4.99%		16.2%	
Model 2: RAC~s (RUE-TN) + s (RUE-TP)	58.1%	+15.8%	18.6%	13.61%	17.2%	1%
Model 3: RAC~s (RUE-TN) + s (RUE-TP) + s (Ratio_NP)	70%	+27.7%	17.9%	12.91%	16.2%	0
Model 4: RAC~s (RUE-TN) + te (Ratio_NP, RUE-TP)	73.3%	+31%	18.3%	13.31%	16.3%	0.1%
Model 5: RAC~s (RUE-TN) + te (RUE-TN, RUE-TP)	73.4%	31.3%	20.9%	15.91%	21%	4.8%

## Data Availability

The data presented in this study are available upon request from the corresponding author.

## Supplementary Materials

The following supporting information can be downloaded at: https://www.mdpi.com/article/10.3390/microorganisms12081685/s1, Figure S1: Residual analysis of optimal model structure in spring (a), summer (b) and autumn (c).
