# Supplementary material for: The Driving Mechanism of Phytoplankton Resource Utilization Efficiency Variation on the Occurrence Risk of Cyanobacterial Blooms
Source: Microorganisms. 2024 Aug 16;12(8):1685. doi: 10.3390/microorganisms12081685 (PMC11356996; doi:10.3390/microorganisms12081685)
Supplement: Supplementary file 1 [file microorganisms-12-01685-s001.zip › microorganisms-3154240-supplementary.pdf]

Supplementary material

Figure S1: Residual analysis of optimal model structure in spring(a), summer(b) and autumn(c).

(a)

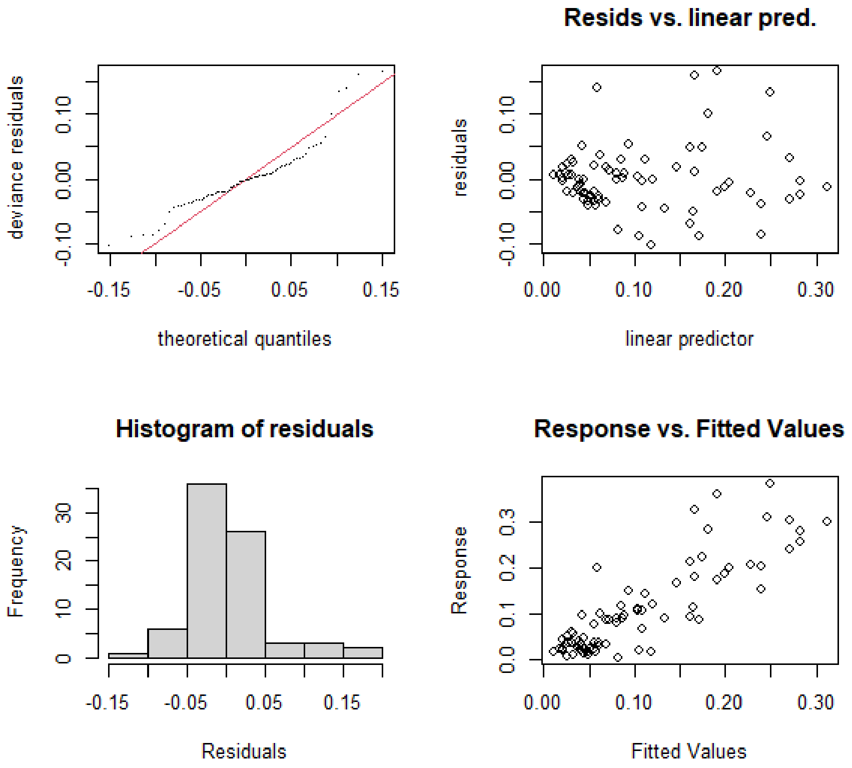

(b)

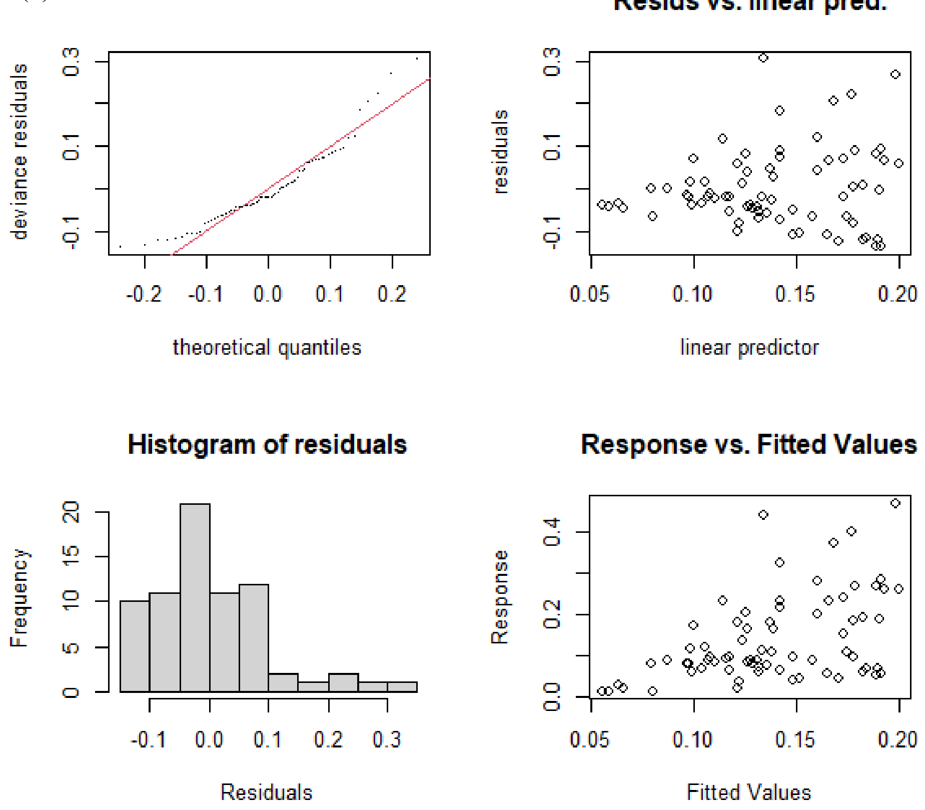
Resids vs. linear pred.
deviance residuals
theoretical quantiles
residuals
linear predictor
Histogram of residuals
Response vs. Fitted Values
Frequency
Residuals
Response
Fitted Values

(c)

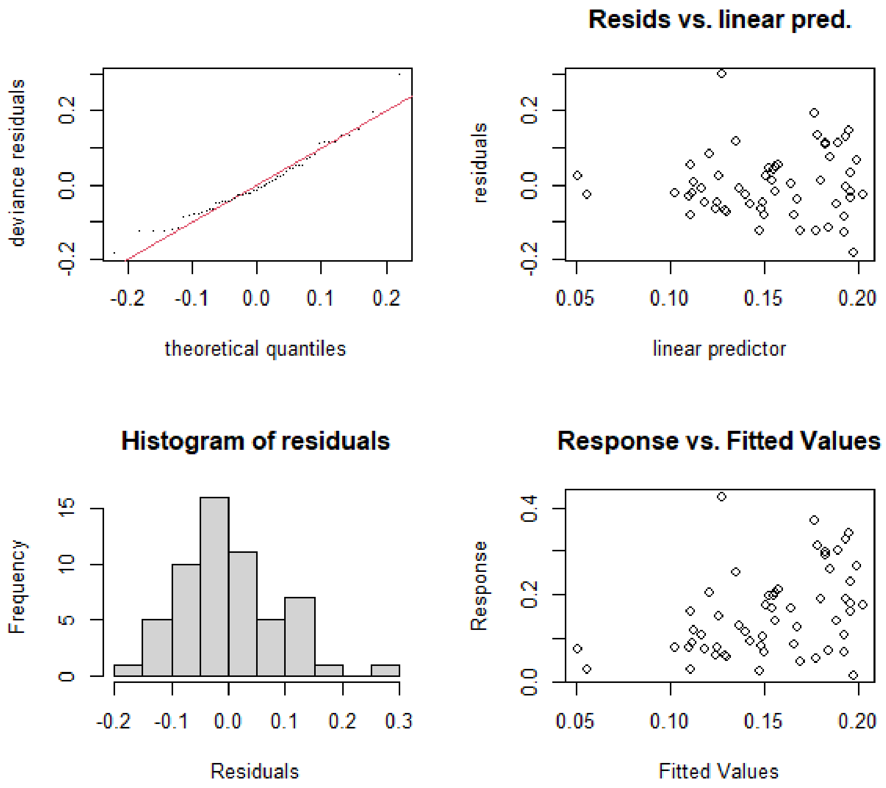
Resids vs. linear pred.
deviance residuals
theoretical quantiles
residuals
linear predictor
Histogram of residuals
Response vs. Fitted Values
Frequency
Residuals
Response
Fitted Values
